# Sodium ions allosterically modulate the M2 muscarinic receptor

**DOI:** 10.1038/s41598-020-68133-9

**Published:** 2020-07-07

**Authors:** Sheli Friedman, Merav Tauber, Yair Ben-Chaim

**Affiliations:** Department of Natural and Life Sciences, The Open University of Israel, Ra’anana, Israel

**Keywords:** Biochemistry, Cell biology, Neuroscience

## Abstract

G protein coupled receptors (GPCRs) play a key role in the vast majority of cellular signal transduction processes. Previous experimental evidence has shown that sodium ion (Na^+^) allosterically modulate several class A GPCRs and theoretical studies suggested that the same also holds true for muscarinic receptors. Here we examined, using Xenopus oocytes as an expression system, the effect of Na^+^ on a prototypical GPCR, the M2 muscarinic receptor (M2R). We found that removal of extracellular Na^+^ resulted in a decrease in the potency of ACh toward the M2R and that a conserved aspartate in transmembrane domain 2 is crucial for this effect. We further show that this allosteric effect of Na^+^ does not underlie the voltage-dependence of this receptor.

## Introduction

G protein-coupled receptors (GPCRs) are the largest superfamily of membrane proteins in the human body and play key roles in human physiology and pathophysiology. The binding of a ligand to a GPCR stabilizes an active conformation of the receptor, which in turn triggers the activation of G proteins, leading to a cascade of cellular responses.

It is now well established that Na^+^ has an allosteric effect on GPCRs. Several studies have shown that Na^+^ modulates the affinity and activity of several GPCRs, including dopaminergic, adrenergic and adenosine receptors^1–3^ (reviewed in Ref.^4^). These biochemical studies were corroborated more recently by structural studies of some GPCRs, such as of the A2a adenosine receptor^5^, the β1 adrenergic receptor^6^ and the δ opioid receptor^7^. These studies provided evidence for the existence of Na^+^ in a defined binding site located in the helical bundle of these receptors, with a conserved aspartate in position 2.50 (Ballesteros and Weinstein numbering^8^) playing a role in Na^+^ binding. Indeed, a mutation in this residue was found to abolish the Na^+^ dependence in several studies^9–12^.

Muscarinic receptors are prototypical class A GPCR family consisting of 5 receptor subtypes (M1R-M5R) that are involved in vast array of physiological processes^13^. Although Na^+^ is not clearly observed in crystal structures of muscarinic receptors^14,15^, molecular dynamics studies predict that Na^+^ may bind to the same binding site also in these receptors as in other GPCRs, and that its binding may modulate agonist binding and receptor activation^16–18^. However, experimental evidence for an allosteric effect of Na^+^ ions on the potency of acetylcholine (ACh) toward the M2R are still lacking.

GPCRs have been shown to be regulated by membrane potential^19–24^ (reviewed in^25^). For two of these receptors, the M1R and the M2R, depolarization was found to induce charge movement, which was suggested to underlie the voltage-dependence of agonist binding^26–29^. Recent studies have proposed that movement of Na^+^ from its binding site is the source of the charge movement associated currents in these receptor and thereby their voltage-dependence^17,18^.

The current study utilizes a functional expression system to test whether extracellular Na^+^ could allosterically affect the M2R and to examine whether this effect underlies the voltage-dependence of this receptor.

## Results

### Extracellular Na^+^ affect the dependence of M2R-mediated GIRK currents on ACh concentration

**Figure 1:**
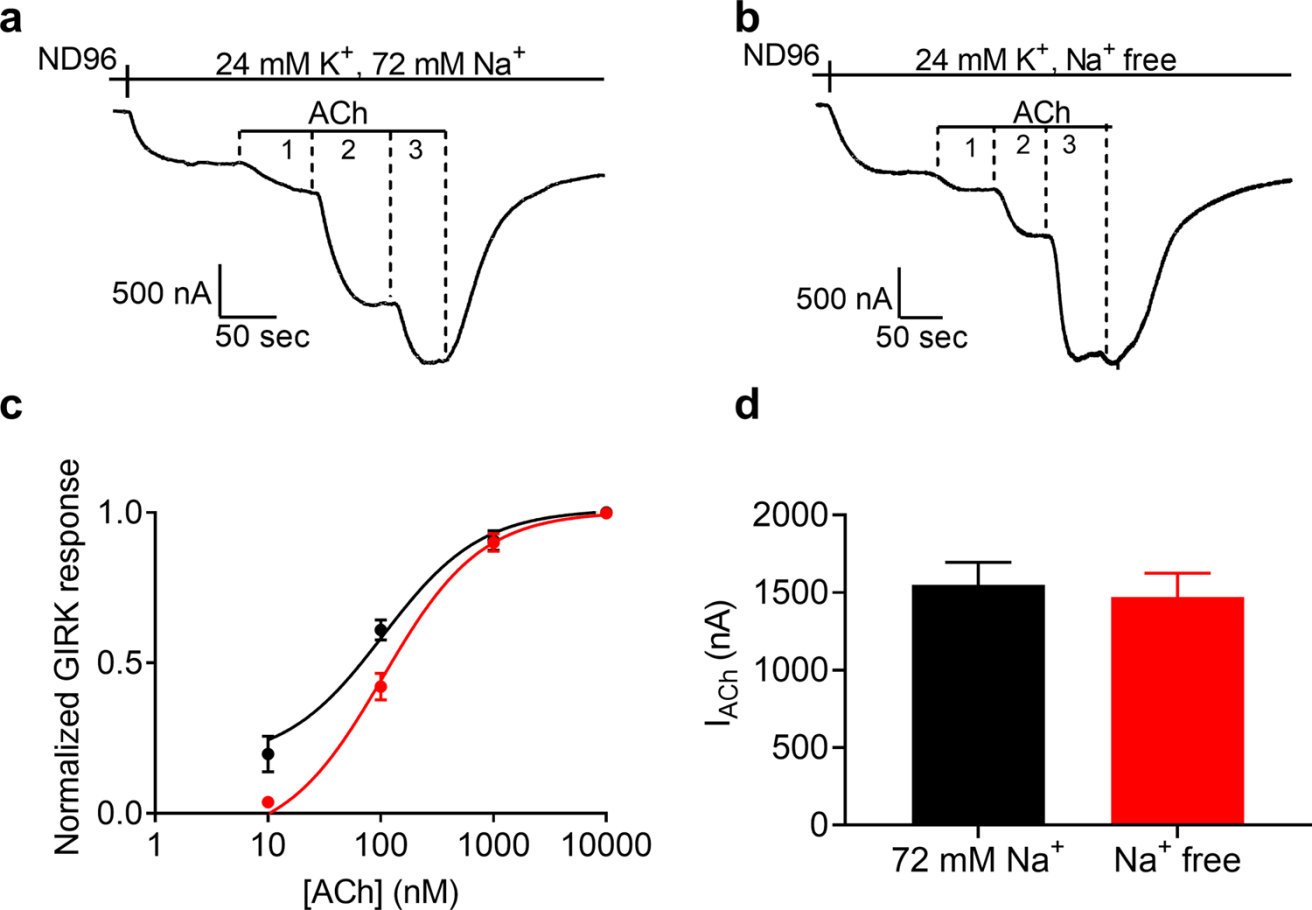
The effect of extracellular Na^+^ on the potency of the M2R. (**a**,**b**) Recordings of ACh-induced GIRK currents in 72 mM Na^+^ solution (**a**) and in Na^+^ free solution (**b**) from the same oocyte. 3 concentrations of ACh were applied sequentially (1, 2 and 3 stand for 10, 100 and 10,000 nM, respectively). (**c**) dose response curves assembled from various experiments conducted at 72 mM Na^+^ solution (black circles) and in Na^+^ free solution (red circles). Each point represents mean (± SEM) from 7–21 oocytes from 6 batches of oocytes. The solid black and red lines were generated by fitting a 3 parameters equation to the data (see “Methods”). (**d**) The maximal amplitude of I_ACh_, evoked by 100 µM ACh in 72 mM Na^+^ solution (black) and in Na^+^ free solution (red). The two bars are not significantly different (unpaired t test, p = 0.71).

To investigate whether extracellular Na^+^ ([Na^+^]_o_) affect the potency of ACh toward the M2R we used, similar to previous studies^19^, *Xenopus Laevis* oocytes as an expression system. Oocytes were injected with cRNAs of proteins involved in the pathway leading to activation of K^+^ currents by M2R via βγ subunits of the G-proteins: The M2R, the two subunits of the GIRK channel (GIRK1 and GIRK2), and the Gαi3 subunit^30^. In this system, the dependence of the ACh-induced K^+^ current (I_ACh_) on ACh concentration (dose–response, DR) was measured in either 72 mM Na^+^ solution or in Na^+^-free solution (Na^+^ was replaced with the large ion N-methyl-d-glucamine, see “Methods”; To ensure full removal of Na^+^ from the extracellular solution, the oocyte was bathed in Na^+^ free solution for at least 5 min prior to the recording). Figures 1a,b depict the experimental protocol for the two levels of [Na^+^]_o_. In each experiment, the oocyte was voltage-clamped to − 80 mV in a low K^+^ (2 mM K^+^) solution, ND96 or ND96-Na^+^ free, and basal GIRK current, I_K_, was developed upon replacement of the ND96 by a 24 mM K^+^ solution, either containing 72 mM Na^+^ (Fig. 1a) or Na^+^-free (Fig. 1b). The two levels of [Na^+^]_o_ were selected in a random order, i.e. first 72 mM Na^+^ and then Na^+^ free or vice versa. Then, 3 concentrations of ACh were applied sequentially (10 nM–10 µM), each leading to an evolvement of currents denoted I_Ach_. The amplitude of I_ACh_ was used as a measure for M2R activation. I_ACh_ was terminated upon washout of the ACh from the bath. Employing this basic experimental protocol with several concentrations of ACh, DR curves at the two levels of [Na^+^]_o_ were constructed. To be able to compare between oocytes at different conditions and across different oocytes we measured fractional I_ACh_. That is, for each [Na^+^]_o_, I_ACh_ at any particular ACh concentration was normalized to I_ACh_ obtained in the same recording at a saturating concentration of ACh.

The results of all of these experiments are depicted in Fig. 1c where DR curves obtained at 72 mM Na^+^ (*black*) and at Na^+^-free solution (*red*) are shown. As illustrated in the figure, the curve shifts to the right when [Na^+^]_o_ is removed from the extracellular solution. Specifically, the EC_50_ is 70 nM in 72 mM Na^+^ and 110 nM at Na^+^ free conditions. The two values are significantly different (p < 0.05). These results suggest that the potency of ACh toward the M2R is allosterically modulated by Na^+^. The removal of Na^+^ from the extracellular solution did not affect the maximal current evoked by the receptor in our experimental system. The average amplitude of I_ACh_ evoked by the maximal ACh concentration at Na^+^ free solution was similar to that obtained at 72 mM Na solution (Fig. 1d, the two conditions are not significantly different; p = 0.74).

**Figure 2:**
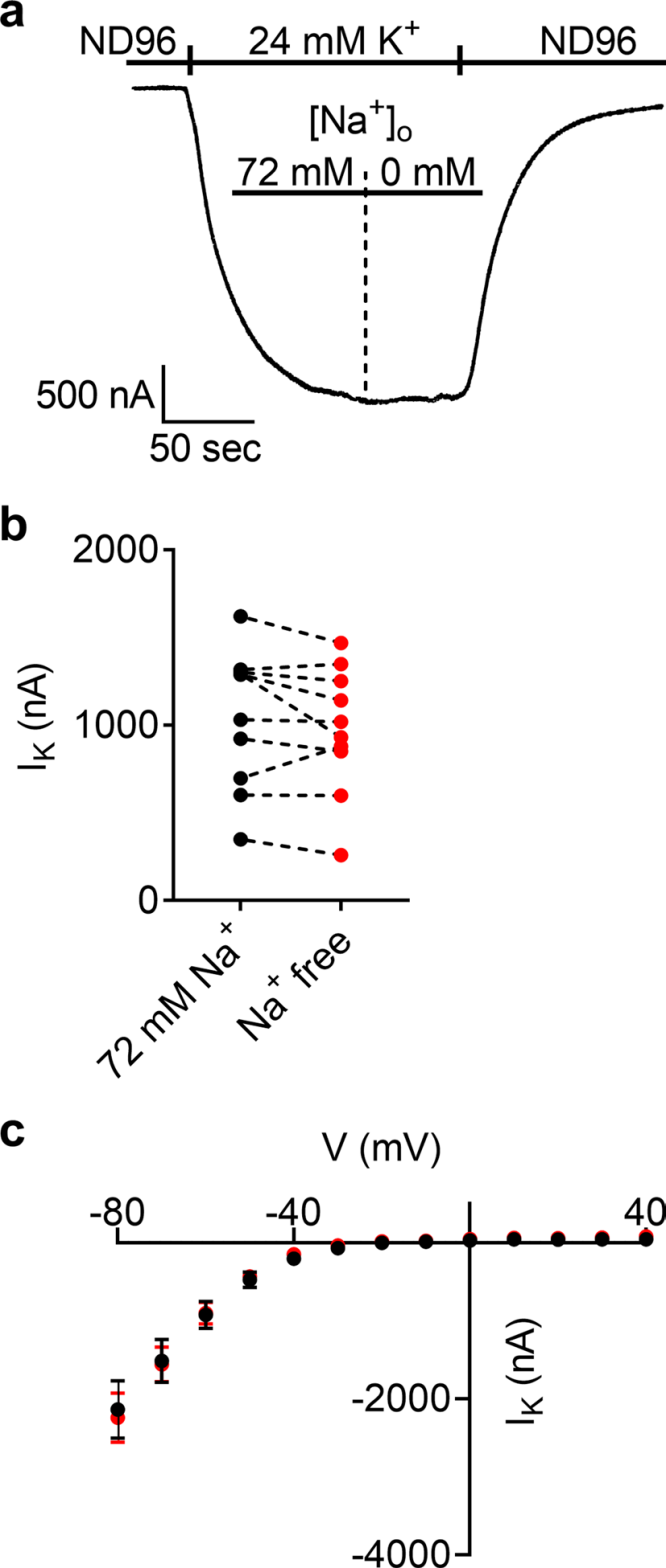
Extracellular Na^+^ does not affect the basal GIRK current (I_K_). (**a**) Recording of I_K_ from an oocyte that expresses the GIRK channel but not the M2R. Removing Na^+^ from the extracellular solution did not affect I_K_. (**b**) Collected results from 10 oocytes. Each two circles connected with a dashed line represent the amplitude of I_K_ from one oocyte at 72 mM Na^+^ (black) and at Na^+^ free solution (red). The order of recordings was selected randomly. Both here and in (**c**), I_K_ was measured by subtracting the current at 24 mM K^+^ solution in the presence of 1 mM Ba^2+^ from the current in its absence. (**c**) Current–voltage relationship of I_K_ at 72 mM Na^+^ solution (black circles) and in Na^+^ free solution (red circles). Results are given as mean (± SEM) from 11 oocytes.

GIRK channels were reported to be modulated by Na^+^^31–33^. To verify that the results of Fig. 1c reflect a genuine effect of Na^+^ on the M2R rather than an effect on the GIRK channels, we measured, in oocytes that express the GIRK channel alone, the effect on [Na^+^]_o_ on the basal GIRK current. This current was proposed^34^ to be due to activation of the GIRK channel by free βγ subunits in the oocyte. Figure 2a shows a representative recording where I_K_ was measured at 72 mM Na^+^ and then at Na^+^ free solution. The figure illustrates that the removal of [Na^+^]_o_ did not affect the amplitude of I_K_. This conclusion was supported by the results of Fig. 2b, where I_K_ was measured first at one of [Na^+^]_o_ and then at the other of [Na^+^]_o_. The order of the recordings was selected randomly. As mentioned above, to ensure full removal of Na^+^ from the extracellular solution, Na^+^ free solution was applied at least 5 min prior to the recording. In these recordings, and in the experiment described in Fig. 2c, I_K_ was measured by subtracting the current amplitude in the presence of 1 mM Ba^2+^ (GIRK channel blocker^35^) from the current in its absence. It was found that in all 10 oocytes tested I_K_ measured in 72 mM Na^+^ was not significantly different from I_K_ at Na^+^ free solution (paired t test, p = 0.16).

To further examine the effect of Na^+^ on the GIRK channels themselves we measured the voltage–current relationship (I–V) of I_K_. To this end, the oocyte was voltage clamped to − 80 mV and the currents after depolarizing pulses to various holding potentials from – 80 to + 40 mV at 10 mV increments were measured in 24 mM K^+^ solution that contained either 72 mM (*black*) and Na^+^ free solution (*red*). In order extract I_K_ from these recordings, the same protocol was repeated in the presence of 1 mM Ba^2+^, and these currents were subtracted from the currents in the absence of barium. The results indicate (Fig. 2c) that [Na^+^]_o_ did not affect either the size or the voltage–current relationship of the GIRK channel. Therefore, the effect of [Na^+^]_o_ on I_ACh_ is likely due to its effect on the M2R.

### Extracellular Na^+^ affect the dissociation of ACh from the M2R

**Figure 3:**
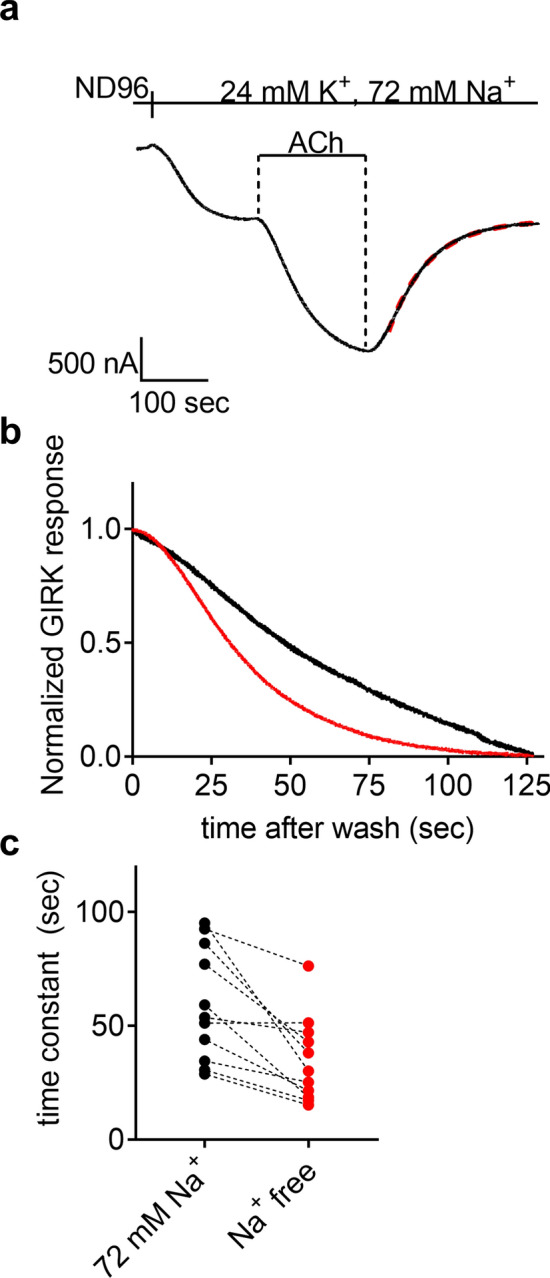
The effect of extracellular Na^+^ on the dissociation of ACh from the M2R. (**a**) The basic experimental protocol. The oocyte was clamped at − 80 mV and I_ACh_ was evoked by application of 1 µM ACh in 24 mM K^+^ solution. Following washout of the ACh with ACh-free solution the current declined and the time constant of the decay was fitted by a standard exponential equation (red dashed line). (**b**) Representative recordings from one oocyte in 72 mM Na^+^ solution (black) and in Na^+^ free solution (red). The currents shown are normalized to enable comparison of the kinetics of the current decline. (**c**) Collected results from 11 oocytes. Each two circles connected with a dashed line represent the time constant of the decay of I_ACh_ following ACh washout from one oocyte at 72 mM Na^+^ (black) and at Na^+^ free solution (red). The order of recordings was selected randomly.

The observed Na^+^-dependent shift in potency of ACh toward the M2R may be due to change in the association rate constant (k_on_) and/or due to an effect on the dissociation rate constant (k_off_) of the ligand from the receptor. Here, we confine our study to k_off_ and examine whether Na^+^ affect the dissociation rate constant. The actual dissociation rate of ACh from the M2R could not be measured directly at our experimental system. However, we have previously shown that the deactivation rate of M2R-induced GIRK currents may serve as a measure for the dissociation of ACh from the M2R^36^. Figure 3a shows a representative recording from one oocyte where the decay of I_ACh_ following the washout of the ACh can be fitted well to a single exponential equation (dashed red line) from which the time constant of the decay can be extracted (see “Methods”). Figure 3b depicts the decay of I_ACh_ following washout with ACh-free solution from the same oocyte at two levels of [Na^+^]_o_. It is seen that I_ACh_ decays more rapidly in Na^+^ free solution (black) than in 72 mM Na^+^ (red). The cumulative results are shown in Fig. 3c. In 9 out of 10 oocytes the time constant of the decay was smaller in Na^+^ free solution than in 72 mM Na^+^, and the mean time constant of the decay at 72 mM Na^+^ was significantly higher than that at Na^+^ free solution (59.1 ± 7.4 and 34.7 ± 5.6 s, respectively) These results suggest that Na^+^ affects the potency of ACh toward the M2R by modulating the dissociation of the agonist from the receptor.

### Asp69 is involved in the effect of Na^+^ on the M2R

**Figure 4:**
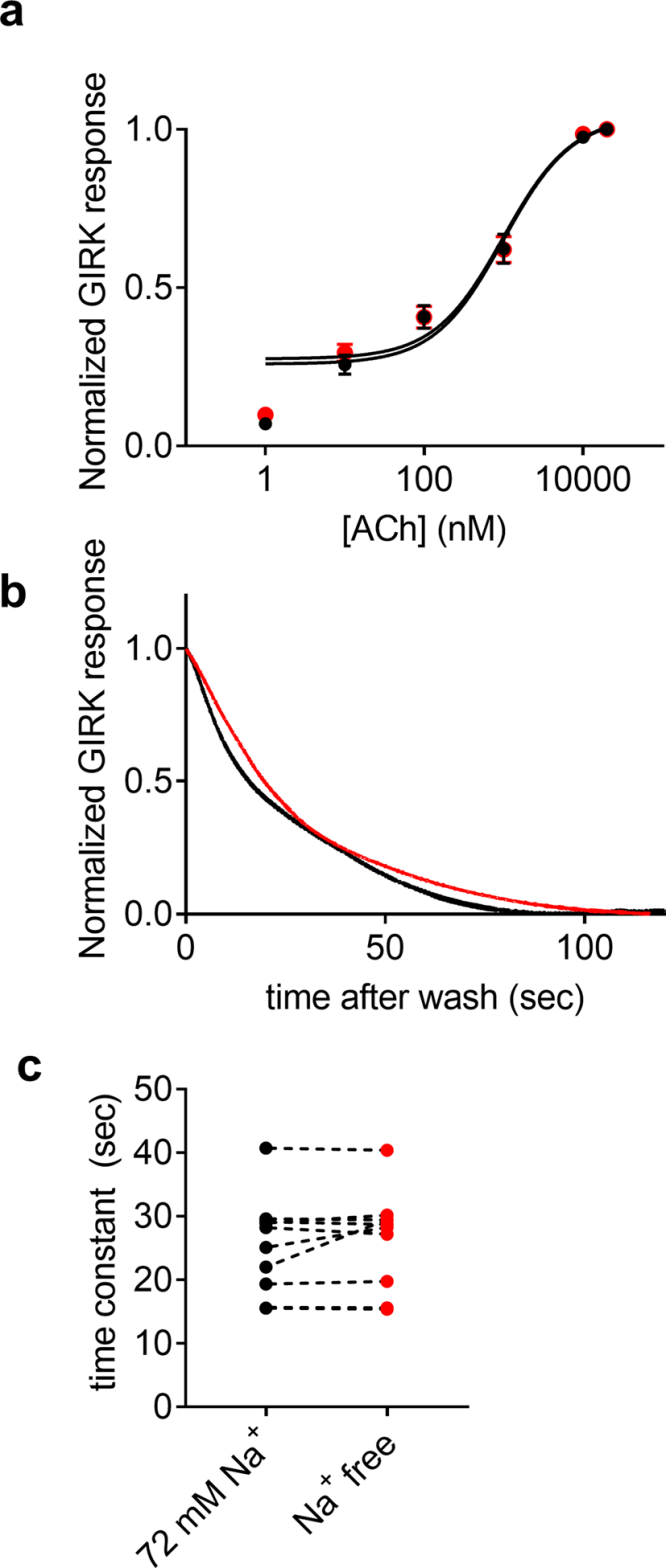
Mutation of Asp69 abolished the Na^+^ dependence of the M2R. (**a**) Dose response curves assembled from oocytes expressing the Asp69ASn mutant at 72 mM Na^+^ solution (black circles) and in Na^+^ free solution (red circles). The number of repetitions for each ACh concentration varied between 7 and 21 from 4 batches of oocytes. The solid black and red lines were generated by fitting a 3 parameters equation to the data (see “Methods”). (**b**) Representative recordings from one oocyte in 72 mM Na^+^ solution (black) and in Na^+^ free solution (red). The currents shown are normalized to enable comparison of the kinetics of the current decline. (**c**) Collected results from 10 oocytes. Each two circles connected with a dashed line represent the time constant of the decay of I_ACh_ following ACh washout from one oocyte at 72 mM Na^+^ (black) and at Na^+^ free solution (red). The order of recordings was selected randomly. The two conditions are not significantly different (p = 0.45).

Several structural and functional studies implicated an aspartate residue in transmembrane domain II (Asp2.50) as being involved in the allosteric effect of Na^+^ on class A GPCRs. These studies suggest that Na^+^ acts via binding at a specific binding site within the helical bundle. Mutating this residue to an uncharged residue results in the abolishment of the Na^+^ dependence in other GPCRs^9–12^ . To validate the role of this residue, Asp69 in the M2R, in Na^+^ binding we mutated it to asparagine (Asp69Asn) and repeated the experiments described in Figs. 1 and 3. The results are depicted in Fig. 4. We found (Fig. 4a), that the potency of ACh toward the Asp69Asn is reduced and it the does not depend on [Na^+^]_o_; As in Fig. 1c, we fitted the data to a three-parameter equation (see “Methods”). Although the fit was not as good as in Fig. 1c (probably due to the complex effect of the Asp69Asn on both agonist binding and G protein activation^37,38^), an EC_50_ values at both Na^+^ concentration could be extracted. The EC_50_ was 920 nM in 72 mM Na^+^ and 1,078 nM at Na^+^ free conditions. The two values are not significantly different (p = 0.66). Corollary to this change in potency, the dissociation of ACh from the Asp69Asn mutant was faster than that of wt M2R, and it was similar at the two levels of [Na^+^]_o_ (Fig. 4b). The collected data from 10 oocytes are shown in Fig. 4c, where the mean time constant of the decay at 72 mM Na^+^ was not significantly different from that at Na^+^ free solution (25.4 ± 2.4 and 26.4 ± 2.4 s, respectively). These results suggest that the dissociation of ACh from the Asp69Asn mutant does not depend on [Na^+^]_o_. These results confirm the role of the Asp 2.50 residue in the allosteric effect of Na^+^ on the function of the M2R.

### Does the allosteric effect of Na^+^ underlie the voltage-dependence of ACh binding to the M2R?

**Figure 5:**
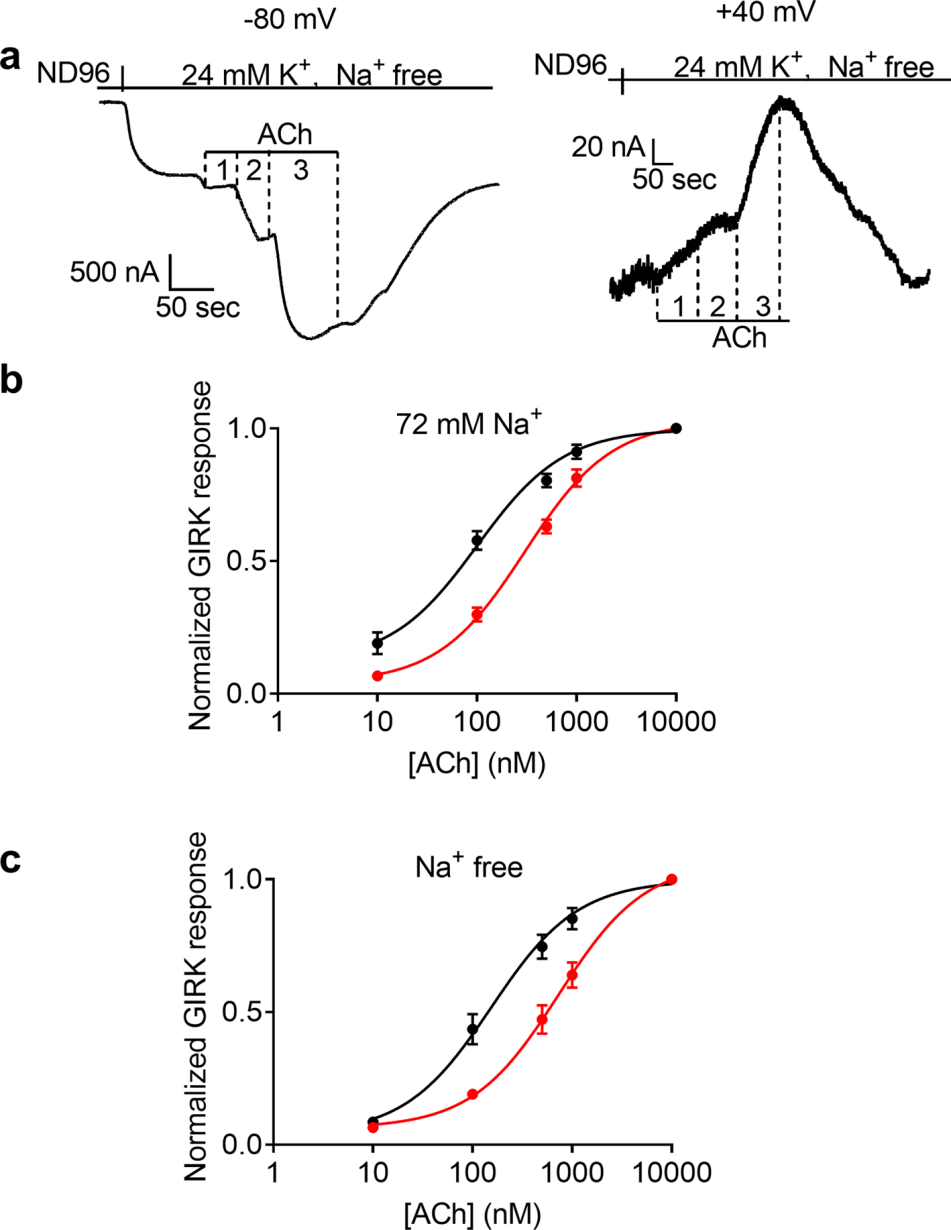
The effect of extracellular Na^+^ on the voltage-dependence of the potency of ACh toward the M2R. (**a**) Representative recordings from one oocyte at − 80 mV (left) and at + 40 mV (right) at Na^+^ free solution. (**b**,**c**) Dose response curves assembled from 5 experiments at 72 mM Na^+^ solution (**b**) and in Na^+^ free solution (**c**). For each oocyte, I_ACh_ was measured at − 80 mV (black circles) and at + 40 mV (red circles) at either Na^+^ concentration, and the response of each concentration was normalized to the response evoked by 10 µM ACh at the same holding potential. Each point represents mean (± SEM) from 8–30 oocytes. The solid black and red lines were generated by fitting a 3 parameters equation to the data (see “Methods”).

We have previously reported that the affinity of the M2R is voltage-dependent; it is higher at resting potential than under depolarization^19^. We further showed that charge movement associated currents are correlated with the change in affinity and may underlie this voltage- dependence^26^. A recent theoretical work proposes that the measured charge movement associated currents may arise from voltage-dependent movement of a Na^+^ from its binding pocket^17^. This movement was suggested to have functional consequences. In light of this, we examined the role of Na^+^ in the voltage-dependence of the affinity of the M2R. To do so, we wished to examine whether the removal of [Na^+^]_o_ affects the voltage-dependence of the M2R. We constructed dose–response curves at two holding potentials, − 80 mV and + 40 mV, as described before^19^. The oocyte was voltage clamp to either − 80 mV or + 40 mV (The order of holding potentials was selected randomly) and the response to three ACh concentrations was measured. Then the oocyte was washed for 10 min in ND96 solution and the experiment was repeated at the other holding potential. Because of the long duration required for the recording at the two holding potential, the dose response relationship was measured for each oocyte either in Na^+^ free solution or in 72 mM Na^+^, and was compared to the dose–response curves obtained from oocytes from the same batches at the other [Na^+^]_o_. Figure 5a shows representative recording from one oocyte at − 80 mV (left) and at + 40 mV (right) at Na^+^ free solution. Dose response curves constructed from recordings obtained from 5 experiments at 72 mM Na^+^ and at Na^+^ free solution are shown in Fig. 5b,c, respectively. The results show that although the potency of ACh toward the M2R is decreased in low [Na^+^]_o_, it remained voltage-dependent to a similar extent; depolarization increased the EC_50_ 3.8 fold at 72 mM Na^+^ and 4.6-fold at Na^+^ free solution.

## Discussion

Na^+^ modulates the affinity and activity of many GPCRs, and the functional role of this phenomenon, as well as its pharmacological potential, is only now begin to be revealed^39^.

Recent advances in the understanding of the structure of class A GPCRs led to new insights into the structural mechanism by which Na^+^ allosterically modulate these receptors^4,5^. This mechanism involves the binding of Na^+^ to a conserved binding site in the transmembrane helices and a collapse of this site following activation of the receptor. This structural mechanism was suggested to account for the observed effect of Na^+^ on the affinity of several GPCRs toward their agonists^4^. Although theoretical studies propose that muscarinic receptors may also be modulated by Na^+^^16,17^, to the best of our knowledge, this has never been demonstrated experimentally for the M2R.

Here, we provide experimental support for this suggestion. We show that the potency of ACh toward the M2R, and specifically its dissociation from the M2R, is modulated by [Na^+^]_o_. Interestingly, our results suggest that Na^+^ increases the affinity of ACh toward the M2R. These results differ from the current notion in the field, where a negative allosteric effect of Na^+^ on agonist binding was reported^16–18^. In particular, two studies with muscarinic receptors suggest the Na^+^ increases the binding affinity of muscarinic antagonist and decreases the binding affinity of muscarinic agonists^40,41^. The first study showed that binding affinity of oxotremorine to cardiac muscarinic receptors is reduced when the tissue was incubated in high Na^+^ concentration^40^. The reason for this difference from our results is not known. It is possible that it is due to the different experimental conditions employed in the two studies. For example, our study used expression system to investigate the M2R in isolation from other muscarinic receptors while Rosenberg et al. studied whole cardiac tissue, where although the M2R is the main muscarinic subtypes, other muscarinic receptor subtypes are expressed^42–44^ and may affect the results. In addition, Rosenberg et al., as well as most other studies that addressed this question, incubated lysed membranes at different Na^+^ conditions, thus allowing the interaction of Na^+^ from both sides of the membrane. It was suggested that intracellular Na^+^ may also haves an effect on the binding properties of GPCRs^45^. Therefore, comparison of the data obtained in such experiments to our data, where only extracellular Na^+^ was modified, may not be adequate.

The second, more recent, study^41^ has reported a mutation of serine residue in position 110 of the M2R with an arginine and suggested that this arginine residue mimics the stabilizing role of the Na^+^. This mutant exhibits increased affinity to two M2R antagonists and reduced the affinity toward the muscarinic agonist iperoxo. These results led to the suggestion that this mutation, and thus, perhaps, also Na^+^, stabilizes the conformation of the whole receptor through tighter inter-helical interactions and therefore increases its affinity toward antagonist and increases it toward agonist. However, their data did not find an effect of Na^+^ alone on the affinity of iperoxo toward the M2R. The source for the inconsistency with our results is not known at this time. It is possible that behavior of the synthetic, high affinity, agonist iperoxo is different from that of ACh. In addition, similar consideration as described above regarding the different interaction site of Na^+^ may also apply here.

Recent simulations^17,18^ suggested that Na^+^ that binds to a specific binding site translocate upon changes in membrane potential, and that these movements may give rise to the gating charges observed for M1R and M2R^26–29^. Other study, however, showed that three tyrosine residues that form a lid above the ligand binding site also contribute to the gating charge^29^. Mutating these tyrosine residues to phenylalanine significantly reduced the measured charge that moves in response to depolarization, although it did not completely abolish it. It is possible that both these mechanisms underlie the measured charge movement, although it is worth noting that charge movement associated currents were recorded in the absence of intracellular and extracellular Na^+^ (but in the presence of NMDG, which may also bind the same binding site). The current study demonstrated (Fig. 5), that the potency of ACh toward the M2R is voltage-dependent even in the absence of extracellular Na^+^. This suggests that the allosteric effect of Na^+^ and the voltage-dependence of the M2R do not share similar mechanism. This conclusion is consistent with recent studies that show that the M2R retain its voltage-sensitivity even when the main Na^+^ interacting residue, Asp69, was mutated^29,46^. Thus, further research is needed in order to fully elucidate the mechanism of voltage-sensitivity of GPCRs.

## Methods

### Preparation of cRNA and oocytes

cDNA plasmids of the two subunits of the G-protein activated inward rectifying K + channel (GIRK) (GIRK1 and GIRK2) and the M2R were linearized with the appropriate restriction enzymes. The Asp69Asn mutant was prepared using Quick-Change II Site-Directed Mutagenesis Kit (Stratagene, La Jolla, CA, USA)^26,29^. The linearized plasmids were transcribed in vitro using a standard procedure^30^.

Xenopus laevis oocytes were isolated and incubated in NDE96 solution composed of ND96 (in mM, 96 NaCl, 2 KCl, 1 CaCl2, 1 MgCl2, 5 Hepes, pH adjusted to 7.5 with NaOH), with the addition of 2.5 mM Na^+^ pyruvate, 100 U/ml penicillin and 100 mg/ml streptomycin^47^. A day after their isolation, the oocytes were injected with the following cRNAs: M2R (200 pg/oocyte) GIRK1 and GIRK2 (200 pg/oocyte for each) and Gαi3.

Materials were purchased from Sigma Israel (Rehovot, Israel).

### Current measurements

The currents were measured 4–7 days after cRNA injection and were recorded using two electrode voltage clamp amplifier^19^ (Warner OC 725C amplifier, Warner Instruments, Hamden, CT). The oocyte was placed in the recording bath containing ND96 solution and was impaled with two electrodes pulled from 1.5 mm borosilicate capillaries (Warner instruments). Both electrodes were filled with 3 M KCl solution. The electrodes resistances were between 0.5 and 2 MΩ. M2R mediated GIRK currents were measured in a 24 mM K^+^ solution (72 mM NaCl, 24 mM KCl, 1 mM CaCl2, 1 mM MgCl2, 5 mM Hepes, pH adjusted to 7.5 with KOH) pCLAMP10 software (Axon Instruments) was used for data acquisition. Basal and M2R- mediated GIRK currents were measured in a 24 mM K^+^ solution (72 mM NaCl, 24 mM KCl, 1 mM CaCl_2_, 1 mM MgCl_2_, 5 mM Hepes, pH adjusted to 7.5 with KOH). In the Na^+^ free solution the 72 mM NaCl was replaced by 72 mM NMDG, and the pH was adjusted with HCl. pCLAMP10 software (Axon Instruments) was used for data acquisition.

### Data analysis

The dose response curves were fitted by the following equation:

$${\text{Y}} = {\text{Bottom }} + {\text{ X}}*\left( {{\text{Top}} - {\text{Bottom}}} \right)/ \, \left( {{\text{EC}}_{{{5}0}} + {\text{ X}}} \right),$$

where Y is the normalized response, X is the concentration of ACh and EC_50_ is the ACh concentration that gives the half-maximal response.

The time constant of the decay of I_ACh_ was extracted by fitting a single exponential to the decay of the current. We began the fit after the current declined to 80% of its maximal level, as described^36^.

### Statistical evaluation

Statistical analysis was done using Prism Graph pad software. Significance was evaluated by Student’s two tailed paired (Figs. 2b, 3c,4c) or unpaired (Fig. 1d) t test.

Estimating the difference between the EC_50_ values was done be the extra-sum-of-squares F test.

### Ethics statement

All experimental procedures used in this study were performed in accordance with relevant guidelines and regulations and were approved by the Hebrew University’s Animal Care and Use Committee (Ethical approval number NS-11-12909-3).
